# Back to the Future: Predicting Individual Tau Progression in Alzheimer’s Disease

**DOI:** 10.21203/rs.3.rs-6772220/v1

**Published:** 2025-06-19

**Authors:** Robin Sandell, Justin Torok, Kamalini G. Ranasinghe, Srikantan S. Nagarajan, Ashish Raj

**Affiliations:** 1 Department of Radiology and Biomedical Imaging, University of California, San Francisco, CA, USA.; 2 Memory and Aging Center, University of California, San Francisco, CA, USA.

**Keywords:** Alzheimer’s Disease, Biophysical Modeling, Statistic Modeling, Precision Medicine

## Abstract

Alzheimer’s Disease (AD) is characterized by the spread of tau neurofibrillary tangles along the brain’s structural network. The marked variability in pathology spread patterns across individuals necessitates a precision medicine approach. Here we introduce Stage-based Network Diffusion (StaND), a novel algorithm that combines statistical staging with biophysical modeling to predict patient-specific tau progression. Using data from 650 subjects in the Alzheimer’s Disease Neuroimaging Initiative, StaND first estimates each subject’s disease stage and then infers their individual tau seeding pattern, agglomeration rate, and transmission rate. The model is applied forward in time to predict regional tau distributions cross-sectionally and longitudinally. StaND outperforms benchmark models in both instances. Inferred tau seed patterns capture spatial heterogeneity, while rate parameters explain temporal and cognitive variability. Despite diverse initial seeding patterns, tau distributions converge across subjects over time. We also identify two distinct tau seeding archetypes with distinct clinical and demographic profiles. StaND offers a powerful new approach for understanding and forecasting the spatiotemporal dynamics of AD and is widely applicable to other neurodegenerative diseases.

## 1 Introduction

Alzheimer’s disease (AD) disease afflicts around 55 million individuals worldwide and is expected to double in the next 20 years [1]. Tau protein neurofibrillary tangles are one of the pathological hallmarks of AD. Tau co-locates with cortical atrophy and is predictive of domain specific cognitive impairments [2–4]. Tau is thought to follow a stereotyped pattern of progression to distinct brain regions characterized by the Braak staging system, originating in the transentorhinal cortex and spreading sequentially to the medial and inferior temporal cortices and associative regions of the neocortex [5]. The Braak stages were developed based on histopathological staining at autopsy and adequately characterize population-level tau spread [6]. However, the advent of *in vivo* positron emission tomography with tau radiotracers (tau-PET) has revealed significant individual variability in tau spread that can deviate from the typical Braak stages. Accurately predicting the spatiotemporal progression of AD in individuals may therefore enable evidence-based prognosis, targeted patient management strategies and the development of precision therapeutic interventions.

Both statistical and biophysical modeling have been applied to characterize and predict tau progression in AD, each manifesting complementary strengths and challenges. Statistical Event-Based Models (EBMs) are a powerful and widely used approach to model the progression of neurodegenerative conditions [7–10]. EBMs assign subjects to disease stages based on a sequential pattern of biomarker abnormalities, transforming cross-sectional data from multiple individuals into a cohort-level timeline of disease progression, thus revealing how pathology unfolds chronologically. EBMs can overcome the paucity of long-term longitudinal data while integrating diverse data types including regional brain atrophy, tau protein accumulation, and cognitive assessment scores. However, EBMs have two significant limitations: as purely statistical methods they lack grounding in the underlying biophysical mechanisms of disease progression, and their population-level insights cannot be extended to individuals. These limitations reduce their utility in precision medicine applications where effective patient care requires mechanism-driven predictions tailored to individuals.

Mechanistically-informed biophysical models have also emerged as potential tools for quantifying spatiotemporal tau progression in the brain [11–16]. These methods are highly complementary to EBMs since they provide crucial mechanistic context for inferred disease trajectories. The Network Diffusion Model (NDM) [17, 18] and its extended version, the Nexopathy in silico (”Nexis”) model, characterize tau progression as a diffusive process between connected brain regions through a system of differential equations. These models have successfully predicted spatiotemporal dynamics of tau spread in mice [19–21]. Network diffusion models assume pathology progression is primarily mediated by the brain’s anatomic connectivity network [22–29]. These models have demonstrated predictive potential in AD [11, 30] and other neurodegenerative diseases [31, 32]. However, biophysical models are by themselves unsuitable as predictive tools for individual subjects because their fitting requires long-term longitudinal data that is challenging to obtain, and they are unable to account for genetic risk, cognitive impairment, and other relevant biomarkers. The key advantage of biophysical modeling is its ability to capture the long-term evolution of tau using mechanistically-informed and interpretable kinetic rate parameters: the rate of tau agglomeration and spread.

We introduce Stage-based Network Diffusion (StaND), a novel framework that combines event-based and network diffusion modeling to predict the full trajectory of tau spread in individuals using only a *single time point* of tau-PET, MRI, and routine clinical-demographic data. StaND consists of three modules. The staging module stratifies subjects into disease stages along a common temporal axis based on their multimodal biomarker profile. The biophysical inference module fits a network diffusion-based model of tau progression along the structural brain network to the subject’s tau-PET data, using the subject’s assigned stage to supply the necessary pathological time scale needed for model identifiability. This fitting process enables the inference of (1) kinetic rate parameters, which capture the speed of disease progression, and (2) the initial seeding pattern, representing the most likely distribution of tau at disease onset that would evolve into the observed pattern via network spread. Finally, the prediction module uses the personalized seed and rate parameters to simulate tau progression forward in time, generating subject-specific predictions of current observed and future unobserved tau distributions. This method of “going back to predict forward” is a unique ability endowed by StaND that sets it apart from all current methods.

We implemented StaND on a multimodal dataset consisting of 650 subjects from the Alzheimer’s Disease Neuroimaging Initiative (ADNI), incorporating cognitive assessments, regional atrophy measurements, and tau-PET imaging. StaND achieved excellent predictability of individual spatiotemporal tau pathology *across the patient’s entire disease course using only single visit data*. We demonstrated substantially higher accuracy than competing benchmark methods. The successful implementation of StaND enables capabilities previously out of reach and uncovers new insights into Alzheimer’s disease progression dynamics. Using StaND we identified the etiologic origins of inter-subject heterogeneity in tau patterning and systematically tracked the temporal evolution of this heterogeneity across disease stages. We report that *most patients do not share a common seeding pattern*. Spatial heterogeneity of tau across subjects follows a distinct temporal pattern: variance initially peaks at seeding sites and progressively diminishes as pathology advances. This progressive convergence of tau challenges the conventional notion of pathologic divergence, both in brain topography and associated clinical symptomatology [6, 17, 33, 34]. We further found that spatial heterogeneity of tau is underpinned by two distinct seed pattern archetypes – focal entorhinal and diffuse temporal lobe seeding. Entorhinal seeding aligns with established AD models, but diffuse temporal seeding represents a previously unidentified pattern. The diffuse temporal seed archetype is associated with clinical indicators of more aggressive pathology and earlier disease onset. Finally, we report that cognitive heterogeneity between individuals is partially captured by the kinetic rate parameters of the fitted StaND model, with higher tau progression rates (faster spreading) correlated to more severe cognitive impairment.

## 2 Results

**Fig. 1:**
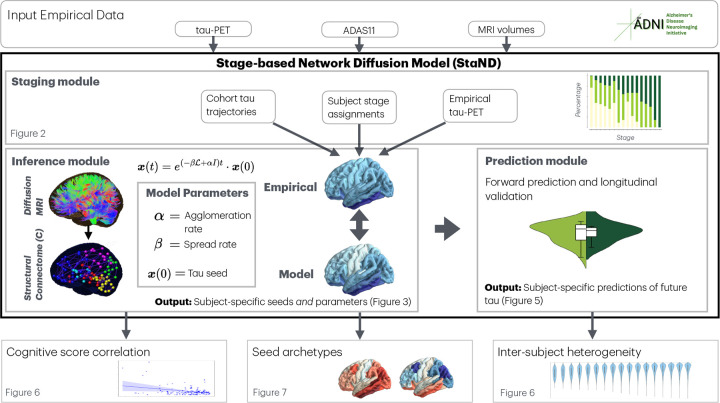
Schematic and flowchart of proposed approach. StaND combines staging of individual subjects with a biophysical inference and prediction module to achieve subject-specific predictions of longitudinal tau spread.

StaND has three components: a) Staging module; b) Biophysical inference module; and c) Prediction module. Each module was thoroughly exercised, tested, and validated, as described in subsections below. A flowchart illustrating each module and its relationship to specific figures of this manuscript is shown in Figure 1.

**Notation**: Scalar variables are denoted by lowercase symbols, vector variables (e.g. regional values of tau) are denoted as lowercase **boldface**, and matrix quantities (e.g. connectivity matrices) are denoted in uppercase. Subscript is used to identify either a single subject (e.g. i) or the entire cohort-average (e.g. c).

### 2.1 The staging module assigns stages to each subject and estimates cohort-level tau trajectories

**Fig. 2:**
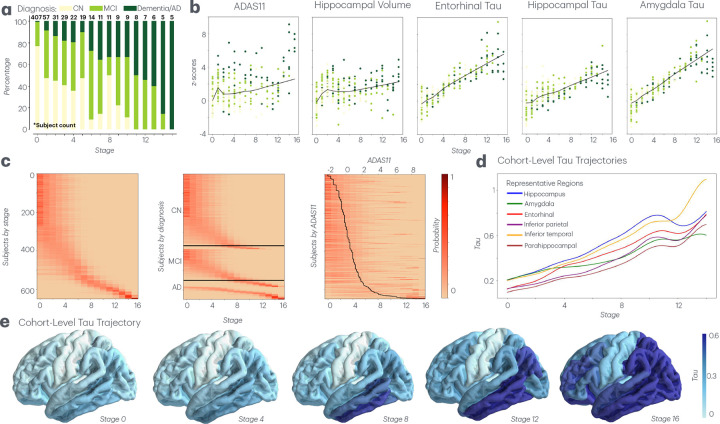
Staging module of StaND. **a.** Histogram of individuals’ most likely stage assignment across diagnostic categories. **b.** Plots of biomarker inputs across stages with lines of best fit indicating monotonic increase. **c.** Heatmaps of each subjects probability distribution across stages, ordered by 1) Most likely stage (low to high); 2) Diagnosis (CN, MCI, AD). Within each diagnostic category ordered by most likely stage; and 3) ADAS11 score (low to high) with a line tracing the ADAS11 score of each subject. **d.** Estimated regional tau distribution across stages in specified regions (Equation (1)). **e.** Estimated regional tau distribution across stages shown on the brain.

StaND first applies a staging module to assign one of 16 disease stages to 650 subjects from stage 3 of the ADNI study. Many iterations of model inputs were tried (Supplemental Figure 1). As shown in Figure 2, StaND’s stage assignments align roughly with diagnostic categories: most CN subjects fall in stages 0−4, MCI in 0−12, and AD/Dementia in 4−16. As expected, all clinical and imaging abnormalities – ADAS11 cognitive score, hippocampal volume, and entorhinal, hippocampal, and amygdala tau – increase monotonically across stages (Figure 2b). Figure 2c illustrates that subjects in early disease stages show broader uncertainty in their stage assignments as indicated by wider probability distributions. Cohort-level tau protein progression follows a predictable trajectory when mapped across disease stages, beginning with accumulation in the amygdala and entorhinal cortex and subsequently spreading to the inferior temporal lobe (Equation 1; Figure 2d,e).

### 2.2 Cohort-level StaND model yields moderate predictions of individuals’ empirical tau

We initiated our biophysical (i.e. network diffusion-based) inference module with the cohort-level tau distribution at stage 0. This approach allowed us to calibrate the model to accurately reflect the stage-wise progression of tau pathology across the cohort. We employed an iterative optimization approach to simultaneously refine both the cohort-level initial seeding pattern $x^{c}(0)$ and kinetic rate parameters $\theta^{c}$. Multiple iterations decreased the overall error from 0.06 to 0.040 and increased Pearson’s correlation coefficient from 0.89 to 0.92 between the staging module’s tau trajectory $y^{c}(t)$ and inference module’s prediction $x^{c}(t)$. Including an L1 regularization term successfully increased the sparsity of the final cohort-level optimized seed $x^{c}(0)$ compared to the original stage 0 seed. Although the iterative refinement of cohort-level seed vectors and rate parameters enabled a strong fit to cohort-level tau trajectories (Supplemental Figure 2a,b), it produced only moderate correlations to individual subjects’ baseline empirical tau at their respective stages (mean R=0.53; Supplemental Figure 2c). This limited correlation indicates that cohort-level modeling alone cannot adequately capture the variability in tau patterning across individuals, underscoring the necessity of personalized modeling approaches.

### 2.3 Subject-specific StaND models yield accurate predictions of individuals’ empirical tau

We next applied the biophysical inference module of StaND to generate individualized models of tau spread. To contextualize the performance of the fully individualized approach, we first present results comparing different algorithmic configurations— whether each inferable quantity is cohort-level or subject-specific – described as model-fitting strategies 1–4 in Section 5.3. Inference of cohort-level seeding and subject-specific kinetic rate parameters achieved a better performance (mean R=0.57) than the entirely cohort-level fitting shown above. This approach uses a single cohort-level seeding pattern for all individuals while allowing StaND parameters – tau agglomeration and spread rate – to vary by individual. The moderate success of this approach suggests that individual variation in tau spread cannot be fully explained by tau progression rate differences alone (Supplemental Figure 3). Inferring subject-specific tau seeding and cohort-level kinetic rate parameters produces a dramatically improved fit to individual subjects’ baseline data (mean R=0.85; Supplemental Figure 4c).

**Fig. 3:**
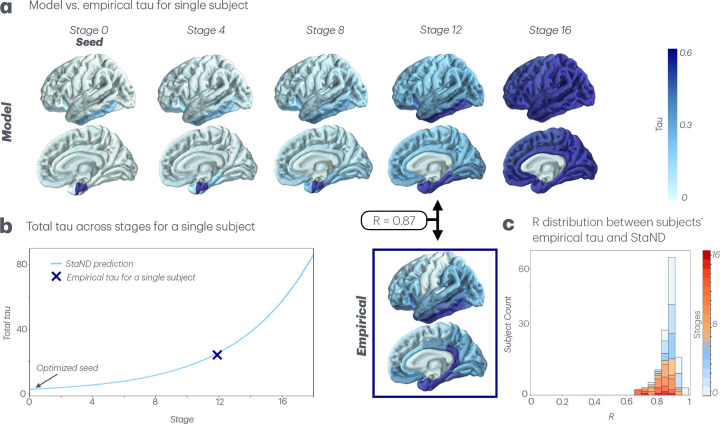
StaND with subject-specific kinetic rate parameters and seeds. **a.** For a single subject, the StaND prediction of tau trajectories vs. their empirical tau. Pearson’s R between model prediction and empirical shown at the subject’s assigned stage. **b.** For the same subject, total tau across stages for both model prediction and empirical tau. **c.** Pearson’s R distribution across all subjects between the model prediction and empirical tau at each subject’s assigned stage, colored by subjects’ stage.

Finally, when all variables (kinetic rates and seeding) are allowed to be subject-specific, the inference module achieved the highest correlation to empirical baseline data (mean R=0.88; Figure 3). Henceforth, all reported results pertain to this final individualized inference approach, which is able to simultaneously account for two critical dimensions of inter-subject variability: spatial and temporal. Incorporating subject-specific seed vectors captures spatial differences in tau’s origin and subsequent progression, while incorporating subject-specific parameters accommodates differences in disease progression rates across individuals. Regions with the highest optimized tau seed across subjects were right entorhinal, left inferior temporal, right inferior temporal, left middle temporal, and left entorhinal cortex (Supplementary Table 1). The superior performance of individualized StaND emphasizes the shortcomings of group-average data that previous trajectory modeling methods like EBMs have employed.

To further compare the individual-level StaND optimization techniques, we calculated the Akaike Information Criterion (AIC) which balances model complexity and fit to provide a metric of overall success (k = the number of inferred quantities being optimized). Subject-specific seed *and* kinetic rate parameter inference is the most complex (k=80) relative to just subject-specific rate parameters (k=2) and just subject-specific seeds (k=78). Despite having more inferred quantities, the joint estimation of subject-specific seeds and rate parameters yielded a significantly lower AIC (8435) compared to using only subject-specific rate parameters (AIC = 33166) or only subject-specific seeds (AIC = 9176). To rule out the possibility that differences in performance were due to varying sparsity of seed tau, we calculated how many regions in each seed contributed to 90% of the total sum or ‘energy’ of the seed as a measure of regional sparsity. Statistical analysis confirmed no significant difference between subject-specific seeds (12.01±6.86 regions, mean ±*SD*) and the cohort-level seed (12 regions, t(164)=0.023,p=0.982). This relationship remained intact at other energy thresholds.

### 2.4 StaND outperforms benchmarks for cross-sectional predictions

**Fig. 4:**
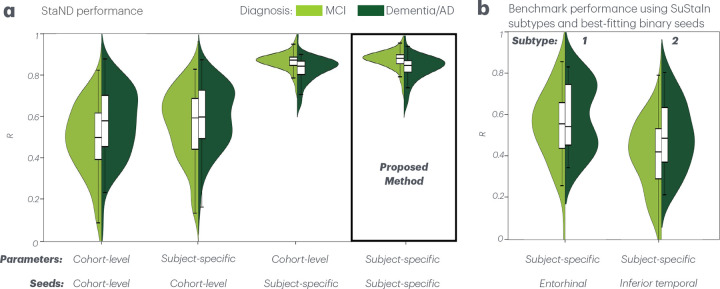
Summary figure comparing StaND optimization strategies to benchmarks: Pearson’s R distributions between model prediction and empirical tau across subjects at each’s respective stage. **a.** Each StaND optimization technique including cohort-level, subject-specific rate parameters with a cohort-level seed, subject-specific seeds with cohort-level parameters, and both subject-specific seeds *and* parameters. Our chosen methodology is highlighted. **b.** Benchmark performance from Vogel et al. (2021): Subject-specific parameters and the best-fitting binary seed region for each of two SuStaIn identified subtypes. Comparison statistics across all methods listed in the main text.

Figure 4 compares each of our StaND inference strategies and benchmark performances. First we show that the individualized prediction model involving subject-specific seeding and subject-specific kinetic rates outperforms other inference strategies (Figure 4a).

For a benchmark comparison, we next used the Subtyping and Staging Inference algorithm (SuStaIn), a type of EBM used by Vogel et al. (2021) [10], to identify two different subtypes in the ADNI3 cohort. Vogel identified four subtypes using SuStaIn, each with a distinct binary regional ”epicenter” for which an Epidemic Spreading Model (ESM), a diffusion-like model, best captured group-level tau spread. Vogel’s subtypes included a limbic subtype (best seeding site: entorhinal cortex), MTL-sparing subtype (middle temporal gyrus), posterior subtype (fusiform gyrus), and lateral temporal subtype (inferior temporal gyrus). Note that our ADNI3 data set was smaller and less diverse than Vogel’s, likely contributing to identification of fewer subtypes in our implementation of SuStaIn. Similarly to Vogel, we ran our inference module with subject-specific parameters to determine the binary seed region that provides the best fit to empirical tau across subjects in each of our two SuStaIn subtypes. Subtype 1’s best-fitting binary seed region was the inferior temporal cortex (mean R=0.44,AIC=47199), somewhat resembling Vogel’s lateral temporal subtype, while subtype 2’s best-fitting binary seed was the entorhinal cortex (mean R=0.55,AIC=92343), resembling Vogel’s limbic subtype (Supplemental Figure 5). Our proposed method – StaND with subject-specific seeds and parameters – dramatically outperformed the benchmark model – each SuStaIn subtype with its best-fitting binary seed (Figure 4b). Note that Vogel et al. reported higher performance metrics for their ESM with best-fitting epicenters (R=0.83,0.51,0.77, and 0.70 across subtypes) than our benchmark values as they used *group-level* analyses which typically yields higher correlations by averaging out subject variability than our *individual-level* results. Paired t-tests between R values of StaND and each SuStaIn subtype with its best-fitting seed regions also confirmed the superior performance of our proposed methodology (subtype 1: t(104)=23,p=0.001); subtype 2: (t(97)=15,p=0.001).

### 2.5 The prediction module accurately forecasts future tau

**Fig. 5:**
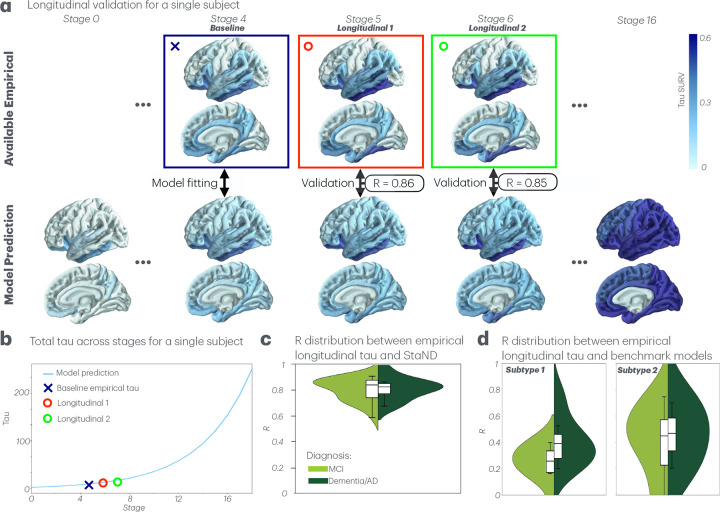
Longitudinal validation of StaND predictions. **a.** A single subject’s StaND prediction vs. available empirical tau data, including baseline and longitudinal follow-ups. Baseline tau is used to fit the model and longitudinal time points 1 and 2 are used to validate its predictive ability. Pearson’s R between the StaND prediction and longitudinal tau is shown at corresponding stages. **b.** For the same subject, total tau across stages for both model prediction and empirical tau (baseline and longitudinal). **c.** R distribution across subjects between StaND prediction and empirical longitudinal tau at corresponding stages. **d.** R distribution between the benchmark models - each SuStaIn subtype with its best-fitting binary seed – and empirical longitudinal tau at corresponding stages.

The prediction module of StaND takes subject-specific seeds and rate parameters and simulates forwards to predict future unobserved tau. StaND’s future predictions correlated well to subjects’ longitudinal data (mean R=0.81; Figure 5c), supporting the model’s ability to accurately forecast individuals’ tau trajectories. Note that the inference module was optimized to fit subject’s baseline data, while longitudinal follow-ups were used only as measures of validation for the prediction module. Finally, our proposed method predicts unobserved longitudinal tau across subjects better than using the best-fitting binary seeds for each of the two SuStaIn subtypes (mean R=0.81 for our proposed method vs. mean R=0.31 and 0.39 for SuStaIn subtypes 1 and 2 respectively; Figure 5d).

### 2.6 Spatial heterogeneity is highest at onset and decreases with disease stage

**Fig. 6:**
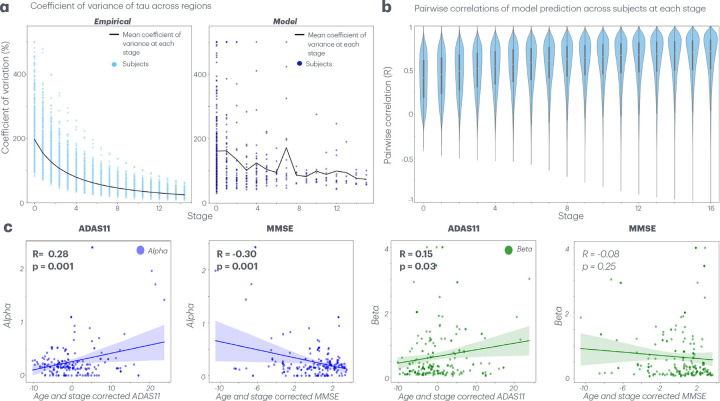
Model based exploration of spatial and cognitive heterogeneity. **a.** Dot plot of the coefficient of variance (CoV) across brain regions for every subject, both their optimized StaND fit (left) and empirical tau (right), shown across stages. Overlaid line plot of the mean CoV across subjects at each stage. **b.** Distribution of pairwise Pearson’s R correlations of StaND predictions across subjects at each stage. **c**. Correlation between model parameters (alpha/beta) and cognitive performance (MMSE/ADAS11). Disease stage and age effects were regressed out of cognitive scores to examine direct parameter-cognition relationships. Tau agglomeration rate (alpha) in blue. Tau spread rate (beta) in green. Statistically significant relationships are highlighted in boldface.

The coefficient of variation (CoV) of tau density across regions decreases with disease stage for both individualized StaND models and empirical tau (Figure 6a). This trend reflects a stage-wise convergence of regional tau distributions, observed in both the mean and spread of CoV across subjects. Figure 6b shows the distribution of pairwise Pearson’s R correlations of tau across all subjects at each stage of their StaND model predictions. The distribution of pairwise correlations increases over stages and time, demonstrating more inter-subject heterogeneity in tau patterning at the origin of pathology than later in disease progression. Because tau vectors are not normally distributed, we repeated the pairwise analysis using Spearman’s correlation, which is distribution-independent, and obtained similar results (Supplemental Figure 6), confirming that differences in pairwise correlations across stages are not driven by vector sparsity.

### 2.7 Individualized StaND kinetic rate parameters capture cognitive heterogeneity

Individualized model parameters – tau agglomeration rate (α) and the tau spread rate (β), show a statistically significant correlation to cognitive scores across subjects. Both parameters increase as cognitive scores worsen. Tau agglomeration rate (α) correlates positively to subjects’ ADAS11 score (R=0.28,p=0.001) and negatively to MMSE score (R=-0.30,p=0.00; Figure 6c). Tau spread rate β similarly increases with ADAS11 score (R=0.15,p=0.03) and decreases with MMSE, albeit not with statistical significant (R=-0.08,p=0.25; Figure 6d). We regressed age and stage out from cognitive score to isolate the relationship between parameters and score. The same relationships and statistical significance remain if neither age and stage *or* each age and stage individually are regressed out.

### 2.8 Two distinct seed archetypes emerge from subject-specific seeding patterns

**Fig. 7:**
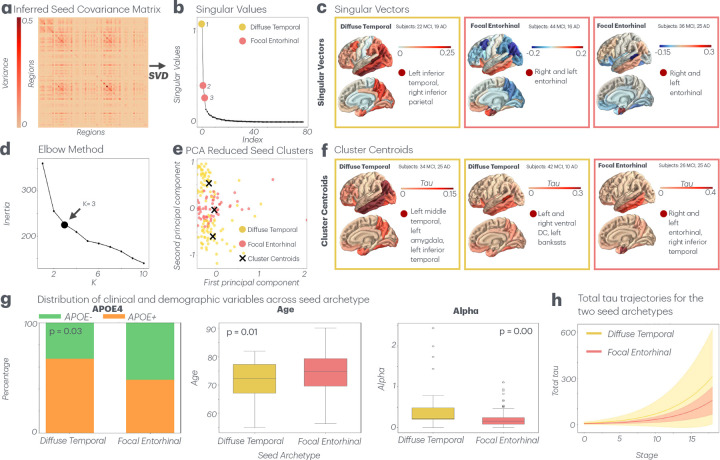
Seed archetypes. **a**. Covariance matrix of subject-specific seeds across subjects. **b.** Singular values of the optimized seed covariance matrix after performing singular value decomposition (SVD), demonstrating a steep decline after the top three components. Top three values highlighted. **c.** Top three singular vectors of the seed covariance matrix (that correspond to the top values) shown on the brain, each of which indicate the most prominent patterns of regional variance in seeds across the cohort. Vectors are color coded by their resemblance to the two emergent archetypes: diffuse temporal and focal entorhinal seeding. Highest and lowest regions listed. **d.** For the K-means clustering analysis of subject-specific seed patterns, a plot of inertia (a measure of cluster coherence) vs. K (the number of clusters) used to determine the optimal K value. We ultimately choose three clusters (K=3). **e.** K-means clusters of subject-specific seed patterns. For plotting purposes, the dimensions of the seed vectors were reduced from 78 to 2 via principal component analysis (PCA). Each subject’s seed is plotted along the top two principal components and colored by its resemblance to one of the two archetypes. Cluster centroids shown in black. **f.** Cluster centroids shown on the brain also color-coded by alignment to emergent seed archetypes. Highest tau regions in each centroid listed. **g.** Clinical, demographic, and model parameter characteristics of diffuse temporal and focal entorhinal seed archetypes. Categorical variables shown as percentage distributions. Continuous variables shown as box plots. **h**. Total tau trajectories for diffuse and entorhinal archetypes across disease stages. Solid lines represent mean values, and shaded regions indicate ±1 standard deviation.

We applied two techniques to explore patterns underlying the heterogeneity of subjects-specific seeds $x^{i}(0)$: singular value decomposition of the covariance matrix and K-means clustering (Figure 7a–f). Both methods revealed the same two underlying archetypes: 1) Prominent distribution of tau seeding density in the entorhinal cortex with low seeding density in other regions and 2) A more diffuse pattern of seeding density primarily across the temporal lobe, but also the frontal and parietal cortices. The seeding covariance matrix is effectively captured by just three singular vectors, with the fourth accounting for only 1.2% of the variance across subjects, allowing us to reduce all seeding heterogeneity into three dimensions. We selected the top three vectors because both their singular values—reflecting the prominence of each variance pattern—and the number of subjects associated with each drop off sharply beyond the third vector; for example, the fourth vector aligns with only 3 out of 165 subjects (Figure 7b). Singular vectors 2 and 3 followed the entorhinal seeding pattern (121 subjects: 80 MCI, 41 AD), and vector 1 followed the diffuse pattern (41 subjects: 22 MCI, 25 AD; Figure 7c). The same coherent subclasses emerged from a k-means clustering analysis of individually optimized seeds. The optimal number of clusters, K, was also three (Figure 7e). Cluster 3 resembled entorhinal seeding (51 subjects: 26 MCI, 25 AD). Clusters 1 and 2 demonstrated a diffuse pattern across the temporal lobe, amygdala, and ventral DC (111 subjects: 76 MCI, 35 AD; Figure 7f).

The identification of distinct seeding archetypes through two independent methods injects further rigor into these conclusions and provides insight into the observed inter–subject heterogeneity at disease onset (Figure 7). Applying our method to a more diverse AD or dementia cohort would likely uncover further tau seed archetypes. Unlike Vogel’s use of SuStaIn, which identifies subtypes based on statistical patterns alone, our identification of seed archetypes has a biophysical basis. Contrary to Vogel, we did not find a clear one-to-one association between focal binary seeding sites and SuStaIn subtypes (Supplemental Figure 5).

The two seed archetypes were also associated with specific clinical and demographic variables. Diffuse seeding is generally linked to earlier onset and more aggressive disease progression compared to focal entorhinal seeding. It is important to note that seed archetype is a static subject-level characteristic and is not expected to vary with measures of disease severity. Rather, it may correlate with fixed disease progression rates or demographic/clinical variables. Subjects with both early and late-stage pathology could have exhibited the same tau patterning at disease onset. As expected, the distribution of MCI versus AD diagnoses across diffuse and entorhinal seed groups was not statistically significant (Chi-square test: $\chi^{2}(1)=2.03,p=0.15$; Supplemental Figure 7b). Similarly ADAS11 scores, a measure of cognitive impairment, did not significantly differ between groups (mean = 13.7 vs. 12.5, One-way ANOVA: $F\left(1,200\right)=\;1.33,p=0.25$; Supplemental Figure 7b). In contrast, APOE4+ carrier frequency, a fixed genetic trait, was significantly higher in the diffuse seed group (Chi-square test: $\chi^{2}(2)=6.08,p=0.04$; Figure 7g). Additionally, diffuse seed subjects were significantly younger than focal entorhinal subjects (mean age = 70.6 vs. 74.0, One-way ANOVA: F(1,200)=7.05,p=0.01; Figure 7g). Within the AD subgroup, this age difference was even more pronounced (mean = 69.8 vs. 75.0). Tau agglomeration rate (α), although a model-derived parameter rather than a clinical variable, was significantly higher in diffuse temporal seed subjects (mean value = 0.44 vs. 0.20, One-way ANOVA: F=19.3,p=0.00; Figure 7g), supporting its role as a proxy for pathological aggressiveness. Conversely, tau spread rate (β) showed no statistically significant relationship to seed archetype (p= 0.21).

The total tau burden trajectory increased more steeply in subjects belonging to the diffuse temporal compared to focal entorhinal seeding archetype (Figure 7h). Finally, each archetype had a distinct tau trajectory originating from its seed origin (Supplemental Figure 8). While the progression rate is higher in the diffuse archetype subjects, the overall spatial patterns between the two archetypes show convergence, mirroring earlier observations (Figure 6a,b).

## 3 Discussion

One of the most challenging unsolved problems in Alzheimer’s Disease research is tracking patients’ multimodal biomarkers over time and predicting their future disease patterns using a single baseline imaging acquisition. Doing so has immense scientific, clinical and diagnostic applicability. Previous statistical approaches using Event-Based Modeling (EBM) are capable of placing single-visit cross-sectional data along a staging-axis but are not suitable for application in individual patients. In contrast, biophysical models of network-based progression, like the Network Diffusion Model (NDM), are applicable for individuals but require longitudinal data to function properly. Here we present a novel solution called Stage-based Network Diffusion (StaND) that can effectively extrapolate patients’ past and future tau patterns, enabling accurate prediction of tau progression across a patient’s entire disease course *using only a single baseline data point*. We reported strong numerical performance for fitting to individual subjects’ baseline tau (mean R=0.88; Figures 3,4), outperforming current benchmark studies [10]. After StaND’s biophysical inference module was used to fit the model to a subject’s *baseline* data, its prediction module was able to accurately forecast the subject’s *future* tau distribution (mean R=0.81; Figure 5).

Fitted model parameters have immediate biological relevance since they capture kinetic rates of protein agglomeration and spread. StaND can also infer etiologic seeding patterns from the subject’s past from single-visit data. StaND achieves this under-constrained yet tremendously useful translational goal by leveraging underlying biophysical mechanisms within the model fitting step. It first infers the subject’s seeding event (”going back”) to then predict future states (”predicting forward”). StaND model-inferred seed patterns successfully capture spatial variability across subjects (Figure 7), while individualized kinetic rate parameters capture temporal and cognitive variability (Figure 6).

StaND can produce individualized models of pathology progression for any neurodegenerative disease characterized by protein spread, and is thus a widely applicable tool. The method of “going back to predict the future” revealed intriguing and previously unreported insights regarding potential sources of spatial heterogeneity and convergent progression of tauopathy, as well as cognitive heterogeneity. These are discussed below.

### 3.1 Spatial heterogeneity of AD tauopathy originates from divergent seed patterns

The StaND biophysical inference module’s ability to infer individual-specific seeding patterns by ”going backwards” in time allowed us to quantitatively explore inter-subject tau variations. It is commonly believed that tau pathology begins at canonical sites in the locus coeruleus and entorhinal cortex [6, 10, 17, 23, 30, 34–36]. These regions have been characterized as a “network epicenter” by Seeley [23, 34], confirmed by Vogel [10], and employed by us in modeling studies [17, 30, 35, 36]. In this view, subsequent individual variations in pathology spread are considered to be dictated by various additional factors like oxidative stress and specific molecules [37]. This ”center-out” hypothesis implies that if we could go back in time, we would find the same or similar seeding sites in all AD patients, while subsequent spread will diverge over time and generate the observed heterogeneity.

However, our results do not support this ”center-out” hypothesis. Instead we found that individually-optimized seeding vectors were highly heterogeneous between subjects, even within the typical-variant mature AD cohort. Neither a single canonical seeding site nor a cohort-level optimized seeding pattern shared across all individuals was able to produce an accurate prediction of individuals’ tau patterns (Supplemental Figure 2c).

### 3.2 Spatial heterogeneity declines as disease progresses

We further uncovered a phenomenon of pathology *convergence* rather than *divergence* across regions and subjects over time. Both inter-subject and inter-region heterogeneity were highest at inferred seeding sites and progressively decreased in later disease stages (Figure 6). A stage-dependent decrease in the coefficient of variance (CoV) across regions in the fitted StaND models is mirrored by *empirical* tau distributions across disease stages (Figure 6a). Inter-subject heterogeneity similarly decreases, demonstrated by stage-dependent increases in pairwise R correlations (Figure 6b).

We conclude that spatial heterogeneity in tau is etiologic in nature, governed by variable seeding patterns beyond previously defined canonical epicenters. This contradicts the traditional ”center-out” paradigm where variation results from divergence from common origins. Rather, the brain’s structural network appears to guide convergence of initially heterogeneous tau distributions. These findings have significant implications for therapeutic approaches, suggesting early interventions may need to target varied seeding patterns rather than uniform spread mechanisms.

### 3.3 Spatial heterogeneity is underpinned by two seeding archetypes with distinct demographic and clinical profiles

Further exploration of the above-noted spatial heterogeneity in tau seeding patterns revealed two distinct archetypes (Figure 7). The first archetype is focal entorhinal seeding, which is consistent with one of the tau ”epicenters” identified by Vogel et al. (2021) and is a classically recognized origin site [6, 10, 23, 30, 35]. In contrast, the second archetype, characterized by diffuse seeding throughout the neocortex with particular prominence in the temporal lobe, challenges established disease models and suggests an alternative pathological pathway that merits further investigation. The emergence of a second previously unidentified archetype strengthens evidence that clinically significant variations in tau seeding and spread exist even within AD variants.

The two tau seed archetypes also align with distinct demographic and clinical profiles across subjects. The diffuse neocortical seeding pattern appears to be associated with more aggressive and early onset disease progression (Figure 7g,h). This is evidenced by several key observations: the diffuse seeding group has a higher prevalence of APOE4 positivity, a lower mean age, higher tau agglomeration rates (α), and more dramatic increases of total tau across stages. The APOE4 allele (a variant of the APOE gene) is a well-established genetic risk factor for AD associated with earlier disease onset and more aggressive progression [38, 39]. Higher rates of APOE4 positivity suggest that a more aggressive AD subtype ensues from diffuse neocortical seeding. The lower mean age of the diffuse seeding group indicates an earlier onset. The consistent relationship between seed archetype and each of these demographic, clinical, and model-inferred variables suggests that the initial distribution of tau pathology may be highly determinant of disease trajectory, lending clinical relevance to our StaND modeling approach. Distinguishing subjects by seed archetype may productively inform the ideal course of treatment and clinical recommendations.

#### 3.3.1 Biophysically inferred seeding archetypes versus SuStaIn subtypes

Our approach to uncovering seed archetypes differs fundamentally from prior attempts to identify subgroups in AD. While Vogel et al. (2021) [10] uses SuStaIn to statistically identify subtypes in their population based on temporal patterns of biomarker progression and then *retrospectively* fits biophysical models to each predefined subtype, we allow subtypes to emerge organically from the biophysical modeling process itself. Our method leverages underlying network diffusion dynamics to naturally reveal distinct pathological trajectories, ensuring that the resulting seed archetypes are grounded in mechanistic principles of tau propagation through brain networks.

A unique contribution from the study by Vogel et al. (2021) was a clear mapping of each subtype to a specific focal, binary seeding site (e.g. entorhinal seeding for subtype 1). Our finding that the AD cohort clusters into two seed archetypes with distinct spatiotemporal trajectories is notable because these archetypes do not conform to focal, binary seeding. When we attempted to use the same focal seeds identified by Vogel on our two SuStaIn-derived subtypes, we did not find a clear one-to-one association, contradicting Vogel’s conclusions (Supplemental Figure 5). We believe therefore that our archetypes –obtained from optimization of tau seed densities across *all* brain regions simultaneously – present more nuanced and complex seeding patterns that better reflect the heterogeneous nature of tau pathology.

StaND’s superior fit to empirical tau-PET data with individualized seeds compared to group-level epicenters demonstrates that substantial heterogeneity in tau spread patterns exists across subjects, not just between the AD subtypes identified by Vogel. Even the well-characterized and clinically less diverse ADNI cohort exhibits meaningful individual differences in pathological progression, supporting the need for precision medicine approaches that account for patient-specific disease trajectories rather than relying solely on group-level generalizations.

### 3.4 Individualized tau progression rates capture cognitive heterogeneity

It is noteworthy that the staging module is unable to fully account for variations in cognitive scores via staging. As Figure 2b,c indicates, ADAS11 remains highly variable even within the same assigned stage. This cognitive heterogeneity appears biological in nature, as prior studies have comprehensively documented correlations between AD biomarker progression and cognition [40]. Our proposed method of achieving individualized fitting of biophysical mechanisms is capable of uncovering and substantiating sources of cognitive heterogeneity.

Individually-fitted StaND parameters – tau agglomeration (α) and spread rate (β) – show a significant relationship to cognitive impairment, especially when age and stage are regressed out. The agglomeration rate represents the speed at which tau accumulates *within* each region, while the spread rate represents the speed at which tau diffuses *across* regions. Agglomeration rate (α) is positively correlated with ADAS11 score (R=0.28,p=0.001) and negatively correlated with MMSE score (R=-0.30,p=0.001), suggesting that higher tau agglomeration rates are intrinsically associated with more cognitive impairment across all ages and stages of disease severity (Figure 6c). The tau spread rate (β) showed a similar, although less pronounced, trend, suggesting a biological mechanism where an increased tau transmission rate across brain regions parallels cognitive deterioration (Figure 6d). The kinetic rate parameters of StaND are therefore predictive of cognitive variability across subjects, even within the same stage. Taken together, these findings highlight that while subject-specific seeds capture spatial variability of tau, subject-specific tau progression rates are needed to capture previously unexplained cognitive variability.

### 3.5 Stage-based network diffusion (StaND): leveraging the complementary power of statistical and biophysical approaches

Event-based models have become popular in recent literature due to their ability to fit longitudinal trajectories at the cohort-level using only cross-sectional data and their inherently multimodal characteristics. We based our staging module off of SuStaIn (Subtype and Stage Inference), which has previously been applied across a range of progressive neurological diseases to disambiguate temporal and phenotypic heterogeneity. Collij et al. (2022) employed Mixture SuStaIn, a similar algorithm that assumes subject’s alignment to multiple subtypes, to stage and subtypes AD subjects based on amyloid beta accumulation [41]. Others applied EBM to non-protein biomarkers. Kudo et al. (2024) used long range and local neural synchrony as inputs to an EBM to subtype and stage AD subjects [9]. Young et al. (2018) replicated the MAPT and GRN mutation genotypes and their distinct patterns of degeneration with SuStaIn’s subtyping and staging based on regional MRI volumes in frontotemporal dementia (FTD) subjects [42]. Vogel et al. (2021) [10] used a network diffusion model to identify tau ‘epicenters’ for each of four SuStaIn subtypes in AD by looping through seed regions. In fact, the ‘epicenters’ they identified align exactly with our top average tau seed regions with the exception of the fusiform gyrus.

However, event-based models cannot be and were not designed to be used as predictive models for individual cases. Furthermore, they do not address the challenge of understanding the mechanistic underpinnings of multimodal (e.g. regional atrophy, amyloid, and cognition) biomarker progression in AD. Event-based models do not directly relate to the underlying biophysical processes involving pathology agglomeration and spread.

Although biophysical models such as the Network Diffusion Model (NDM) and related spread models like SIR [43] are highly expressive for time-evolving pathology and biophysically informed, they also present challenges as predictive models. First, they lack a natural means of accommodating mediating factors such as genes, demographic variables, cognitive assessments, and environmental determinants. A key variable used in network spread models is the patient’s time since disease onset (”t”), which is unknown from available longitudinal data in patients who may have had onset decades earlier. Finally, individual subject measurements typically span only 1–4 years, making it impossible to obtain long-term dynamic trajectories of disease necessary for proper fitting of model kinetics.

To address the challenges posed by each technique, we employed our innovative hybrid modeling approach, Stage-based Network Diffusion (StaND). First, we applied a multi-factorial staging module to stage subjects along a common disease axis. This ”pseudo-time” becomes a measure of time since onset while cross-sectional data is transformed into a long-duration ”pseudo-longitudinal” time series suitable for fitting biophysical models. Next, we fit the network diffusion-based inference module to cohort-level tau trajectories outputted by the staging module and used this fitted version as a base model on which to build individually-customized predictors, such as subject-specific seeding sites and subject-specific kinetic rate parameters. In this manner, the resulting model implicitly benefits from multimodal biomarker progression captured by event-based modeling while still giving biophysically-informed subject-level predictions.

Our *individual* tau predictions (mean R=0.88; Figure 4) showed a better fit to subjects’ empirical baseline data than *group-level* predictions reported by Vogel et al. (2021) (R=0.83,0.51,0.77, and 0.70 for the four SuStaIn-derived subtypes). StaND also outperforms non-network diffusion modeling approaches for predicting tau spread. Schoonhoven et al. (2023) applied a susceptible-infected (SI) model to predict group-level tau spreading patterns from functional connectivity patterns (R=0.584 at preclinical stages and R=0.38 for AD) [44]. Franzmeier et al. (2020) demonstrated that functional connectivity patterns predict future tau accumulation in AD using a tau-weighted connectivity model, achieving a group-level performance of R=0.50 (ADNI) and R=0.42 (BioFINDER) [3]. Karlsson et al. (2025) developed machine learning models using multimodal biomarkers to predict individual-level tau burden in temporal regions only (R=0.81-0.83), while our StaND method achieved superior individual-level *brain-wide* tau prediction [45].

### 3.6 Limitations

Our works faces several limitations. First, non-zero tau in controls (likely due to off-target binding) led to high, diffuse stage 0 tau densities that were biologically implausible. We mitigated this by including an L1 regularization term in our StaND loss function to encourage seed sparsity. Further staging module refinements would help address this limitation. Our cohort included only typical AD and MCI, appropriate for exploring heterogeneity within the classic spectrum but lacking other variants (logopenic, behavioral) that contribute to disease diversity—work currently ongoing in our laboratory. While our longitudinal validation supports StaND’s predictive power, available ADNI data spans at most 4 years, a small fraction of disease progression. Future work should assess StaND’s predictions against longer time intervals as additional data becomes available. A detailed comparison to purely machine learning approaches was outside the scope of this work but will be pursued in future projects.

## 4 Conclusion

Our proposed Stage based Network Diffusion model (StaND) represents a significant methodological advance in tau modeling, achieving individual-level prediction accuracy that outperforms existing approaches — from network diffusion models and functional connectivity-based predictions to machine learning — by jointly leveraging statistical and biophysical techniques. Our approach also provides novel insight into the source of temporal and spatial heterogeneity in AD tau pathology.

## 5 Online Methods

### 5.1 Data

#### Patients and Data Sources.

Data used in this study was obtained from the Alzheimer’s Disease Neuroimaging Initiative (ADNI3) database. Empirical AV1451-PET (‘tau-PET’) imaging data, regional atrophy based on T1-weighted MRI, and cognitive scores, were obtained from a sample of 650 patients consisting of 64 AD, 196 mild cognitive impairment (MCI), and 390 healthy controls (CN). Longitudinal data existed for 267 subjects, 136 of which are MCI or AD. Our analysis used only cross-sectional data and excluded patients from ADNI who had incomplete data across the relevant biomarkers. Patients ages range from 55–90 years and include 215 females and 435 males.

#### Brain regional parcellation and tau co-registration.

High-resolution T1-weighted sagittal MR images of patients’ brains were obtained from the ADNI website (https://adni.loni.usc.edu/data-samples/access-data/). These images were acquired using the 3D MPRAGE sequence with specific parameters: an 8-channel coil, TR (Repetition Time) of 400 ms, minimum full TE (Echo Time), 11 degrees flip angle, slice thickness of 1.2 mm, resolution of 256 × 256 mm, and a FOV (Field of View) of 26 cm. Briefly, the ADNI image processing pipeline was as follows. The T1 images underwent normalization to the Montreal Neurological Institute (MNI) space and segmentation using SPM8’s unified co-registration and segmentation scheme (https://www.fil.ion.ucl.ac.uk/spm/). Grey matter (GM) was divided into 86 regions of interest (ROIs) based on the Desikan-Killiany (DK) atlas [46]. Post-processed tau-PET (AV1451) images were retrieved from ADNI and underwent a series of further processing steps. Initially, these images were normalized to a common space, adjusted using cerebellar values, and re-sliced to ensure uniform voxel resolution. To establish a reference point, PET images from healthy controls were utilized to generate an average image, which was then normalized to the MNI space using SPM8’s linear and non-linear transformations. This transformation was consistently applied to all individual PET images, aligning them with the same resolution as the 86-region DK atlas. Finally, mean AV1451 values were computed for each of the GM ROIs to quantify tau-PET signals within specific brain regions. Tau-PET data is available for download from the ADNI website. The PET data was then transformed into regional group statistics.

#### Diffusion MRI (dMRI) and tractography processing.

All processing was carried out within a custom pipeline based on the NiPype framework [47]. T1 images were segmented into gray (GM), white matter (WM), and CSF tissue maps using SPM, where T1 images were registered and transformed to MNI space. dMRI volumes were corrected for Eddy currents and small head movements by registering the diffusion-weighted volumes to the first non-diffusion-weighted volume via an affine transformation using FSL FLIRT [48]. Skull-stripping was performed using FSL BET. Details regarding this processing can be found in a previous publication [49].

#### Structural connectivity network

Different structural connectivity networks were constructed using the same DK parcellations as above. First, we obtained publicly available dMRI data from the MGH-USC Human Connectome Project (HCP) to create an average template connectome. The HCP contains data from 418 healthy brains [50]. Subject-specific structural connectivity was computed on dMRI data as described in Abdelnour et al. [51] and Owen et al. [52]. *Bedpostx* was used to determine the orientation of brain fibers in conjunction with FSL FLIRT [48]. Tractography was performed using *probtrackx2* to determine the elements of the adjacency matrix. 4,000 streamlines were initiated from each seed voxel corresponding to a cortical or subcortical GM region and the number of streamlines reaching a target GM region was recorded. The weighted connection between the two structures, c(i,j), was defined as the number of streamlines initiated by voxels in region i that reached any voxel in region j, normalized by the sum of the source and target region volumes. The average connection strengths between both directions, (c(i,j) and c(j,i)), formed the undirected edges. To determine the geographic location of an edge, the top 95% nonzero voxels were computed for both edge directions and the consensus edge was defined as the union between both post-threshold sets. Details regarding this processing can be found in Verma et al [53].

### 5.2 Staging-based Network Diffusion (StaND) Module 1: Staging

The staging module of StaND probabilistically assign stages to subjects on a common disease continuum based on the abnormality of relevant AD biomarkers. Events are defined as the progression of a biomarker past predetermined severity thresholds. Stages are the midpoint between consecutive events. Traditional event-based models (EBMs) first infer the temporal sequences of events and then assign a stage to each subject that aligns with their biomarker values. The output of the EBM is a probability distribution for each subject across stages as well as a most likely stage at which the probability of assignment is highest. EBMs have previously been applied to stage and subtype subjects with progressive neurodegenerative conditions using biomarker inputs of amyloid beta, tau-PET, regional atrophy, and neural synchrony measures [10, 41, 42, 54]. Our staging module of StaND is based on Z-score Subtype and Stage Inference algorithm (SuStaIn) [7, 42]. However, our approach diverges from SuStaIn in two ways. 1) SuStaIn assigns subtypes as well as stages to each subject. Instead of subtyping subjects up front, we assumed one subtype and then applied individual-level network diffusion models to explore heterogeneity in the cohort. 2) In contrast to predetermined abnormality z-scored thresholds of SuStaln, we estimated the abnormality thresholds for each biomarker by maximizing the MCMC sampling likelihood of the data.

The staging module receives an input feature array with dimensions of the number of biomarkers x the number of severity thresholds for each biomarker. The number of features becomes the number of stages along the shared pathology axis. The number of input features must therefore be balanced with the number of samples, in our case 650 ADNI3 subjects [10]. We selected 5 biomarkers and 3 abnormality thresholds per biomarker, totaling 15 features and 16 stages (Supplemental Figure 1). We applied the same rule as Vogel et al. (2021) of enforcing approximately 20 samples per feature. The consequence of an imbalanced number of features for the sample size is a sparse and uneven distribution of subjects across stages, which skews the longitudinal interpolations of tau progression (Supplemental Figure 1).

We selected hippocampal volume, ADAS11 cognitive score, and entorhinal, hippocampal, and amygdala tau density as our biomarker inputs. We experimented with over 20 combinations of biomarkers, including frontal, temporal, and occipital lobe volumes, MMSE scores, and tau densities in the parahippocampal gyrus, inferior parietal, and inferior temporal lobes. Our final biomarker selection was justified by three key criteria: 1) The staging module’s effectiveness at assigning appropriate stages to each diagnostic category (ensuring controls were not placed in advanced stages), 2) Confirmation that the interpolated biomarker progression across stages showed the expected monotonic increase, and 3) Each selected biomarker’s well-documented relevance to Alzheimer’s disease progression in the established literature. More details on selecting biomarker inputs to the staging module are included in **Supplemental Results** and **Supplemental Figure** 1.

The staging of subjects is sensitive to the abnormality thresholds set for each biomarker. Appropriate z-score abnormality thresholds were selected by maximizing the MCMC sampling likelihood as performed by Kudo et al. (2024) [54]. For each set of thresholds, we ran 1,000,000 MCMC sampling iterations. The combination of thresholds for each biomarker that produced the highest data likelihood was ultimately selected after an exhaustive set had been assessed.

First, we normalized tau-PET signals by using the cerebellum as a reference region to create Standardized Uptake Value Ratios (SUVRs). The cerebellum was selected because it typically remains relatively unaffected by tau pathology, even in advanced Alzheimer’s disease. Specifically, we calculated the average signal between the left and right cerebellar cortex for each subject, divided each regional tau value by this average, and subtracted 1 from the ratio to center the data. Next, we performed age regression to eliminate confounding effects of age. We implemented linear regression using scikit-learn’s LinearRegression model with default parameters (including intercept) on each brain region separately. For each region, we regressed out the effect of age by calculating residuals (observed values minus model predictions). Sex was not included as a covariant in this regression model. Finally, we calculated z-scores for each age-regressed regional value with respect to the healthy control cohort.

#### From stages to tau trajectories

To infer longitudinal tau progression from cross-sectional data, we created interpolated cohort-level tau trajectories across stages using each subject’s stage assignments from the staging module. For each subject i in our dataset, we obtained 1) the most likely stage of disease progression $\left(s^{i}\right)$ based on their biomarker profile and 2) a distribution of the likelihood of that subject belonging across all possible stages, given by pr(i=s). A subject’s final stage assignment $s^{i}$ is the peak of their probability distribution. Tau pathology distributions were expressed as an 86 × 1 vector y of Standardized Uptake Value Ratio densities (SUVR) in each brain region, corresponding to the 86-region DK brain atlas. Due to the known off-target binding behavior of the tau-PET tracer to striatal regions [55], data from these regions was excluded for comparisons of model predictions to empirical data. Excluding a total of 9 striatal regions yields a 78-region tau vector. In the proceeding methods, y always represents the ground truth tau distribution.

Using all of the information outputted by the staging module we calculated a cohort-level tau map, y(s), at every stage s by taking a weighted sum of each subject’s empirical tau map $y^{i}$ (Equation (1)). Weighting by the likelihood of a subject’s assignment at each stage pr(i=s) served as a smoothing mechanism for the resulting interpolation and overcame the uneven distribution of subjects across stages. The resulting cohort-level tau trajectories were used to fit the inference module of StaND.


(1) $$y(s)=\frac{\sum_{i}\text{pr}(i=s)y^{i}}{\sum_{i}\text{pr}(i=s)}$$

### 5.3 StaND Module 2: Biophysical inference

#### 5.3.1 Extending the Network Diffusion Model

The spread of disease-causing pathological protein species through the brain’s network (connectivity matrix, C) over time t can be modeled as a diffusion process starting from a seed location, using the Network Diffusion Model (NDM) introduced by Raj et al. (2012) [17]. A Network Diffusion Model assumes that pathology transmission from region 1 to region 2 is a first-order diffusion process along the fiber projections. We denoted model-predicted pathology in all DK regions r as an 86 × 1 vector x. Tau pathology over time was defined as x(t)={xr(t)∀r∈[1,N]}. The following above equation represents the diffusive process on the whole brain network:

(2) $$\frac{dx(t)}{dt}=-\beta ℒx(t)$$

(3) $$ℒ=I-D^{-1/2}CD^{-1/2}$$

where ℒ is the graph Laplacian, D is a diagonal matrix containing the degree (total outgoing connections) of each node, I is the identity matrix, and β is a kinetic rate parameter that controls how fast tau spreads between regions, known that the ”spread” or ”diffusion” rate. Equation (2) allows a closed-form solution $x(t)=e^{-\beta ℒt}x(0)$ where x(0), referred to as the ”seed vector,” represents the initial pattern of tau across regions at disease onset t=0. The matrix exponential $e^{-\beta ℒt}$, or the diffusion kernel, acts as a spatial and temporal blurring operator on x(0). The model’s diffusion time t is in arbitrary units (a.u.).

In this study, we extended this basic model by adding a key process: protein accumulation and/or clearance modeled via first-order kinetics:

(4) $$\frac{dx(t)}{dt}=(-\beta ℒ+\alpha I)\cdot x(t)$$

where α is a kinetic rate parameter that controls how fast tau is produced by the seeding and templated corruption of healthy tau, or the ”agglomeration rate.” StaND parameters α and β will be collectively denoted by the global parameter set θ. Below is the closed form solution to StaND in which x(t|α,β,x(0)) is the model prediction of tau at time t given kinetic rate parameters α and β and tau’s origin x(0).


(5) $$x(t|\alpha ,\beta ,x(0))=e^{(-\beta ℒ+\alpha I)t}x(0)$$

##### **Initial seeding pattern**.

The model assumes that the initial distribution of misfolded tau is given by the vector x(0). The canonical implementation of this initial pattern is typically given by a focal seeding site (e.g. entorhinal cortex), in which case the vector x(0) will be zero in all regions except for the seeding site. However, in this study we will not enforce a pre-specified focal seeding site. Instead the most appropriate seeding pattern will be inferred from either cohort or individual-level model-fitting, as described below.

#### 5.3.2 Estimating the time axis

Fitting the biophysical inference module requires a longitudinal time axis to capture tau throughout the full course of disease progression. In previous applications of network reaction-diffusion-type models in mice, this time axis was provided by known time intervals between tau seeding and each data collection point [19, 21]. However, in humans the time since disease onset is unknown when patients come into the clinic for tau-PET scans and cognitive testing. This is where the inclusion of the staging module provides a critical innovation. Our staging module supplies a temporal axis along which every subject is staged in order to fit the inference module. Here we use stages *s* as pathologically-relevant ”pseudo-time” instead of chronological time t. Note that in doing so we assume a linear relationship between stages and time specified as

(6) t=as+b,

which when substituted into Equation (5) gives

$$x(s|\alpha ,\beta ,x(0))=e^{\left(-\beta ℒ+\alpha I)(as+b\right)}x(0)$$

With a simple redefinition of model parameters: $\alpha \leftarrow a\alpha ,\beta \leftarrow a\beta ,x(0)\leftarrow e^{-b(\beta L+\alpha I)}x(0)$, we obtain

(7) $$x(s|\alpha ,\beta ,x(0))=e^{(-\beta L+\alpha I)s}x(0)$$

The “new” kinetic rate parameters and seeding vector are still unknown constants and must be estimated from empirical stage-resolved tau trajectories. The linear relationship between stage and time is reasonable given our focus on estimating progression on the axis of disease severity rather than chronological time. However, further work should explore the nature of this relationship in more depth and consider non-linear relationships.

#### 5.3.3 Overview of StaND model fitting

Both the global parameters θ and the tau seed vector that initializes the model x(0) can be optimized to fit the model to empirical data at any number of time points. The ill-posed nature of simultaneous optimization of both seed vectors and global parameters of StaND necessitated a systematic two-stage optimization approach to develop individual spatiotemporal models of tau spread. Four logical strategies were implemented and evaluated for individual-level model optimization:

1. **Cohort-level StaND inference**. We first attempted cohort-level optimization, where both the seed vector and parameters were shared across all subjects. This required an iterative process between global parameter and tau seed optimization.
2. **Subject-specific StaND parameter inference**. This approach uses a single cohort-level seeding pattern $x^{c}(0)$ for all individuals while allowing StaND parameters – tau agglomeration and spread rate – to vary by individual. We experimented with various different plausible cohort-level seed vectors, including the stage-0 tau pattern, specific seeding sites like the entorhinal cortex and hippocampus, and finally the iteratively-optimized cohort-level seed vector.
3. **Subject-specific StaND seed pattern inference**. This approach uses cohort-level StaND parameters – tau agglomeration and spread rate – while optimizing individual seed vectors.
4. **Subject-specific StaND seed *and* parameter inference**. We finally assessed the case when both seed vector and StaND parameters are allowed to be subject-specific. This strategy became our final methodology. The end product is an algorithm for individual fitting and prediction that captures all the observed heterogeneity observed between subjects. Note that the multitude of parameters in this technique can be prone to over-fitting to the noise in individuals’ data in certain cases. Use of subject-specific seed patterns with cohort-level parameters may be appropriate when applying this method in future investigations. A cohort-level seed can act as a regularizer, constraining the solution space. The subject-specific differences in kinetic rates and speed of transmission are also accounted for by the EBM-derived stage for that subject.

For all of the above techniques, we tried a variety of optimization methods to ensure the global minima of the parameter space was reached, including basin hopping, differential evolution, Powell, and L-BFGS-B algorithms. We then took the smallest error value across all methods for each optimization process. The cost function for StaND optimization is as follows:

(8) $$ℰ(x,y)=\|y-x{\|}^{2}-0.5(ℛ(x,y))$$

in which x is the model prediction, y is empirical data, and ℛ is the Pearson’s correlation coefficient.

For tau seed optimization we introduced an L1/L2 regularization term, $\frac{\lambda \|x(0){\|}_{1}}{\|x(0){\|}_{2}}$, to promote seed sparsity without altering the scale of the seed. Seed sparsity is both more biologically plausible and helps to ensure that the model optimizes a non-zero diffusion rate β. The optimal λ was determined by plotting the L1 cost term and MSE score and selecting the ”elbow” of the plot at which both are minimized.

The four levels of model inference summarized above are described in detail below. Detailed pseudo-code for all algorithms that implement these four strategies can be found in **Supplemental Methods**.

##### 1. Cohort-level StaND inference

For the first optimization strategy, we fit the model to the cohort-level tau trajectories $y^{c}(s)$ outputted by the staging module by iterating between parameter and tau seed optimization. The product of this iterative process was parameter and seed values that best represented the entire cohort. We began the iterative model fitting process by optimizing the model parameters $\theta^{c}$ to fit the longitudinal cohort-level tau trajectories from staging module $y^{c}(s)$ (1). The tau seed $x^{c}(0)$ was set to be the s=0 vector from the staging module’s interfered tau trajectories. Cohort-level model parameters $\theta^{c}$ were calculated by minimizing our cost function, as shown below. Here EBM tau trajectories $y^{c}(s)$ serve as an ”empirical” ground truth.


(9) $$\theta^{c}=\underset{\theta }{\text{arg}\;\text{min}}\left\{ℰ\left(y^{c}(s),x^{(}s|\theta^{c},x^{c}(0)\right)\right\}$$

Next, we applied the optimized cohort-level parameters $\theta^{c}$ from equation (9) as fixed parameters for StaND and optimized the tau seed $x^{c}(0)$ to fit the staging module’s tau trajectories $y^{c}(t)$, as shown below.


(10) $$x^{c}(0)=\underset{x(0)}{\text{arg}\;\text{min}}\left\{ℰ\left(y^{c}(s),x\left(s|x(0),\theta^{c}\right)\right)+\lambda \frac{\|x(0){\|}_{1}}{\|x(0){\|}_{2}}\right\}$$

After the first round of parameter optimization, we implemented an iterative refinement process. We alternated between fixing the previously optimized seed vector $x^{c}(0)$ from Equation (10) while optimizing the global parameters $\theta^{c}$ and then fixing those optimized parameters while refining the seed vector. This back-and-forth iteration between parameter and seed optimization progressively improved the StaND fit to the staging module-inferred tau trajectories $y^{c}(s)$, ultimately yielding optimal cohort-level parameters $\theta^{c}$ and seed vector $x^{c}(0)$.

##### 2. Subject-specific StaND parameter inference

To model the tau progression of *individual* subjects’, we optimized the model to fit each subject i’s empirical tau vector $y(s^{i})$ at their assigned stage $s^{i}$. We first tried optimizing the tau progression rate parameters for each individual while maintaining a fixed cohort-level seed. To do so, we initiated StaND with the cohort-level seed $x^{c}(0)$ from Equation (10) and optimized individual parameters $\theta^{i}$ for every subject i, shown below.


(11) $$\theta^{i}=\underset{\theta }{\text{arg}\;\text{min}}\left\{ℰ\left(y\left(s^{i}\right),x\left(s^{i}|\theta ,x^{c}(0)\right)\right\}\right.$$

##### 3. Subject-specific StaND seed pattern inference

In our next model optimization strategy, we maintained the previously optimized cohort-level parameters $\theta^{c}$ as constants. We then individually optimized each subject’s seed vector $x^{i}(0)$ to match their the empirical tau pattern observed at their assigned stage $s^{i}$ (Equation (12)). Individually optimized seeds $x^{i}(0)$ represent the initial tau distributions for which the model simulation best aligns with each subject’s empirical baseline. By estimating an individual-level seed pattern we are first ”going back in time” to then forward simulate a subject’s current and future tau.


(12) $$x^{i}(0)=\underset{x(0)}{\text{arg}\;\text{min}}\left\{ℰ\left(y\left(s^{i}\right),x\left(s^{i}|x(0),\theta^{c}\right)\right)+\lambda \frac{\|x(0){\|}_{1}}{\|x(0){\|}_{2}}\right\}$$

##### 4. Subject-specific seed *and* StaND parameter inference

For our final approach, we started with the individually optimized seed patterns $x^{i}(0)$ for each subject from Equation (12) and inferred subject-specific parameters with Equation (11). This involved optimizing each subject’s kinetic rate parameters while keeping their unique seed vectors fixed. The final individually fitted models $x^{i}(t)$ represent the evolution of tau over the full course of the disease of each subject i. Based on the success of each model fitting strategy, we selected this one for the proceeding analysis. For all StaND inference module fitting strategies, we excluded control subjects and participants assigned to stage 0. No meaningful diffusion modeling can be performed at this stage because the optimized seed vector is identical to the empirical tau pattern with no actual diffusion having occurred. The final subject count after excluding controls and stage 0 subjects is 104 MCI and 61 AD subjects.

### 5.4 StaND Module 3: Prediction and longitudinal validation

Having fitted a unique set of kinetic rate parameters and seeding pattern to each individual subject’s baseline tau-PET scan and other imaging and non-imaging biomarkers, we are now in a position to perform useful predictions about the subject’s future tau patterns. Here we approximated each subjects’ stage at longitudinal follow-ups by assuming a 1 to 1 linear relationship between Δt in years and stage Δs, given that the average length of AD progression is approximately the number of stages in our model (Equation 6). The stage of each longitudinal time point $s_{l}$ was determined by adding the known Δt between the baseline and longitudinal follow-up visits to the staging module’s estimated stage at baseline $s_{b}$ for each subject. Using subject-specific StaND parameters and seeding, we predicted future tau patterns $x\left(s_{l}|\alpha^{i},\beta^{i},x^{i}(0)\right)$ for each subject by evaluating equation (7) at $s=s_{l}$.

We correlated this individually optimized StaND prediction to empirical longitudinal tau-PET data to validate the model’s ability to predict future tau distributions. Only a subset of 297 ADNI3 subjects have longitudinal tau-PET data, 136 of which are MCI or AD, ranging from 1 to 3 follow-up visits within a maximum window of 4 years from baseline testing. Pearson’s correlation coefficient R and mean squared error (MSE) were calculated across all longitudinal follow-up visits for each subject.

### 5.5 Characterization of seed archetypes

The individualized spatiotemporal tau trajectories $x^{i}(t)$ generated by our Stage-based Network Diffusion model (StaND) facilitated the analysis of subject-specific tau origins. To characterize the underlying patterns within these heterogeneous seed distributions, we employed two complementary techniques.

We first calculated a covariance matrix $A=\sum_{i}x^{i}(0){\left(x^{i}(0)\right)}^{T}$ of individually optimized seed vectors $x^{i}(0)$ across all subjects and performed its singular value decomposition (SVD):

(13) $$A=U\Sigma V^{T}$$

Matrix $U=\{u_{k}\}$ contains the singular vectors of the covariance matrix, and matrix Σ contains singular values that correspond to each vector. Singular vectors reveal the fundamental patterns of variance in individually optimized seeds $x^{i}(0)$, with their corresponding singular values quantifying each pattern’s contribution to overall cohort variance. We focused on the three most prominent patterns $\left\{u_{1},u_{2},u_{3}\right\}$(the singular vectors with the highest singular values) and classified each subject by determining which of these top vectors most strongly correlated with their optimized seed pattern.

Our second technique for characterizing underlying patterns across subjects was K-means clustering of the subject-specific seeds. We tried multiple clustering algorithms, often in conjunction with dimensionality reduction, on our data, including UMAP + HBDSCAN clustering, spectral clustering, and K-means. K-means was the only algorithm to successfully differentiate more than one primary seed pattern. We selected the optimal number of clusters K at the elbow of a plot of inertia vs. K, where inertia serves a measure of cluster quality and internal coherence. Inertia is defined as the sum of the squared distances of subject seed patterns to their closest cluster centroid. The top three singular vectors and cluster centroids both represent underlying archetypes across subject-specific seeds.

## Supplementary Files

This is a list of supplementary files associated with this preprint. Click to download.

- SupplementalMaterial.pdf

## Data Availability

We plan to share all relevant data (regional tau, atrophy and amyloid, cognitive scores) and source code publicly upon paper acceptance, via our GitHub repository (https://github.com/Raj-Lab-UCSF). Original MRI and PET images may be obtained directly from ADNI.
